# Hippocampal functional connectivity across age in an *App* knock-in mouse model of Alzheimer's disease

**DOI:** 10.3389/fnagi.2022.1085989

**Published:** 2023-01-12

**Authors:** Zachery D. Morrissey, Jin Gao, Liang Zhan, Weiguo Li, Igor Fortel, Takaomi Saido, Takashi Saito, Arnold Bakker, Scott Mackin, Olusola Ajilore, Orly Lazarov, Alex D. Leow

**Affiliations:** ^1^Graduate Program in Neuroscience, University of Illinois at Chicago, Chicago, IL, United States; ^2^Department of Psychiatry, University of Illinois at Chicago, Chicago, IL, United States; ^3^Department of Anatomy & Cell Biology, University of Illinois at Chicago, Chicago, IL, United States; ^4^Department of Electrical and Computer Engineering, University of Illinois at Chicago, Chicago, IL, United States; ^5^Preclinical Imaging Core, University of Illinois at Chicago, Chicago, IL, United States; ^6^Department of Electrical and Computer Engineering, University of Pittsburgh, Pittsburgh, PA, United States; ^7^Department of Bioengineering, University of Illinois at Chicago, Chicago, IL, United States; ^8^Department of Radiology, Northwestern University, Chicago, IL, United States; ^9^Laboratory for Proteolytic Neuroscience, RIKEN Center for Brain Science, Wako, Japan; ^10^Department of Neurocognitive Science, Institute of Brain Science, Nagoya City University, Nagoya, Japan; ^11^Department of Psychiatry and Behavioral Sciences, Johns Hopkins University, Baltimore, MD, United States; ^12^Department of Neurology, Johns Hopkins University, Baltimore, MD, United States; ^13^Department of Psychiatry, University of California, San Francisco, San Francisco, CA, United States; ^14^Department of Computer Science, University of Illinois at Chicago, Chicago, IL, United States

**Keywords:** Alzheimer's disease, functional connectome, interhemispheric, resting-state functional magnetic resonance imaging (rs-fMRI), *App* ^*NL-G-F/NL-G-F*^, hippocampus, excitation-inhibition balance, hyperexcitability

## Abstract

**Introduction:**

Alzheimer's disease (AD) is a progressive neurodegenerative disease. The early processes of AD, however, are not fully understood and likely begin years before symptoms manifest. Importantly, disruption of the default mode network, including the hippocampus, has been implicated in AD.

**Methods:**

To examine the role of functional network connectivity changes in the early stages of AD, we performed resting-state functional magnetic resonance imaging (rs-fMRI) using a mouse model harboring three familial AD mutations (*App*^*NL-G-F/NL-G-F*^ knock-in, APPKI) in female mice in early, middle, and late age groups. The interhemispheric and intrahemispheric functional connectivity (FC) of the hippocampus was modeled across age.

**Results:**

We observed higher interhemispheric functional connectivity (FC) in the hippocampus across age. This was reduced, however, in APPKI mice in later age. Further, we observed loss of hemispheric asymmetry in FC in APPKI mice.

**Discussion:**

Together, this suggests that there are early changes in hippocampal FC prior to heavy onset of amyloid β plaques, and which may be clinically relevant as an early biomarker of AD.

## 1. Introduction

Alzheimer's disease (AD) is the most prevalent form of dementia. In a small percentage of cases, however, patients that carry specific mutations in the amyloid precursor protein (*APP*), presinilin-1 (*PSEN1*), or presinilin-2 (*PSEN2*) genes will develop an autosomal dominant form of AD known as familial AD (FAD) (Campion et al., 1999; Bateman et al., 2012; Cruchaga et al., 2012; Long and Holtzman, 2019). However, most cases of AD are sporadic of unknown cause (Hampel et al., 2021). It is thought that AD develops decades before cognitive manifestations (Jack et al., 2013), making it difficult to understand the early processes that occur in the disease. Thus, identifying early biomarkers of AD would be important for both understanding the progression of the disease as well as for clinical translation.

Previous studies have suggested that disrupted connectivity in the default mode network, in particular the hippocampus, is one of the early functional changes in AD (Greicius et al., 2004; Allen et al., 2007; Li et al., 2018), leading to the network degeneration hypothesis, which proposes that pathology begins in select vulnerable regions, leading to synaptic loss and dysfunction that then spreads to other anatomically-related brain regions (Delbeuck et al., 2003; Seeley et al., 2009; Oh et al., 2015). In the hippocampus, there are also differences between the left and right hemispheres. These include differences in receptor expression, physiology, and spatial memory, among other features (Jordan, 2020). Furthermore, interhemispheric connectivity is important for some cognitive tasks, which has been shown to be defective in AD patients (Lakmache et al., 1998), and has been shown to act as a potential compensatory mechanism, e.g., after sleep deprivation (Zhu et al., 2016). However, it is still not fully understood how functional connectivity (FC) changes across age, and if there are asymmetric differences in those changes, particularly in the context of AD.

Here, we used *in vivo* resting-state functional magnetic resonance imaging (rs-fMRI) to investigate changes in hippocampal connectivity across age in wild-type and *App*^*NL-G-F/NL-G-F*^ knock-in (APPKI) mice harboring the Swedish, Arctic, and Beyreuther/Iberian mutations in the amyloid precursor protein (*App*) gene, which results in the production of A*β* plaques at approximately 4 months of age and leads to learning and memory deficits starting at approximately 6 months of age (Saito et al., 2014). In contrast to transgenic mouse models of FAD, this model does not encounter potential artifacts due to overexpression of one or more transgenes (Saito et al., 2014).

We observed strengthening in interhemispheric connectivity across age, but this effect was weaker in the APPKI mice relative to the wild-type. Conversely, we observed reduced intrahemispheric connectivity with age. However, this reduction was not symmetric, with the right hemisphere showing a greater decline with age relative to the left hemisphere, and differences between wild-type and APPKI specifically in the right hemisphere. Together, these results help to improve our understanding of the early functional changes that occur in the hippocampus in mice with AD pathology, and how FC is affected by age.

## 2. Materials and methods

### 2.1. Animals

C57Bl/6 wild-type (WT) and *App*^*NL-G-F/NL-G-F*^ knock-in (APPKI) (Saito et al., 2014) female mice were used in this study. Animals were fed *ad libitum* and housed in standard housing cages on a 12 h light-dark cycle. All methods were approved by the UIC Institutional Animal Care and Use Committee. The number and age of mice used is shown in Supplementary Table 1.

### 2.2. Magnetic resonance imaging

Mice were scanned *in vivo* using a 9.4 T Agilent MRI system (Santa Clara, California, USA) in early (4 months), middle (10 months) and late (>15 months) age groups. Mice were anesthetized using 1–2% isoflurane, secured using a bite bar head mount to restrict head motion, and the respiratory rate and ambient temperature were monitored using an SAII gating and monitoring system for small animals (SA Instruments, NY) while scanning. *T*_2_-weighted images were acquired using a fast spin echo sequence (TR = 2,000 ms, *TE* = 10 ms, echo train length = 8, slice thickness = 1 mm, number of slices = 20, slice gap = 0 mm, FOV = 19.2mm × 19.2 mm, matrix size = 128 × 128, acquisition time = 2 min 12 s).

Two coronal slices at the hippocampus were selected (approximately –2 and -3 mm posterior from bregma) for resting-state functional MRI (rs-fMRI). Prior to acquisition of the rs-fMRI images, shimming was applied in a region (7.5 × 10.5 × 5 mm) that has the selected slices at the center to mitigate local field inhomogeneity. Resting-state fMRI images were acquired using an echo planar imaging sequence (*TR* = 3,000 ms, *TE* = 10 ms, slice thickness = 1 mm, number of slices = 2, slice gap = 0 mm, FOV = 19.2 × 19.2 mm, repetitions = 200, acquisition matrix size = 64 × 64, reconstruction matrix size = 256 × 256, acquisition time = 40 min 16 s).

### 2.3. Functional MRI preprocessing

The first three volumes were discarded to account for any artifacts from initiating the scanning sequence. SPM12 was used to apply motion correction to align to the first volume to account for any head motion. The CONN batch interface was used to perform slice-timing correction, Art outlier detection, smoothing (0.2 mm3 Gaussian kernel), and bandpass filtering (0.008–0.09 Hz).

#### 2.3.1. Coregistration

**Figure 1:**
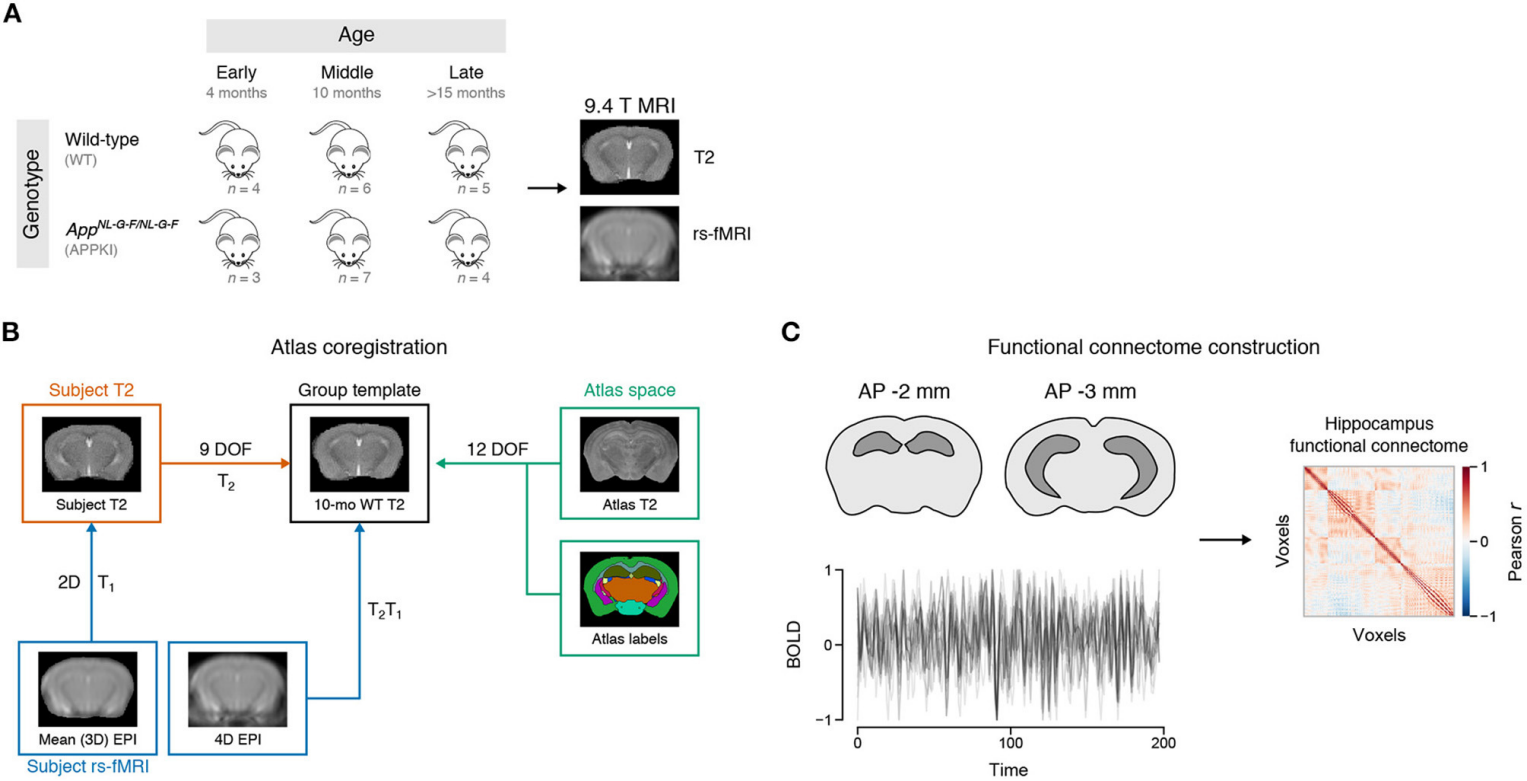
Experiment design. **(A)** Schematic of MRI timeline. Female mice were scanned at early (4 months), middle (10 months), and late (>15 months) age using a 9.4 T Agilent MRI system. WT: Wild-type. APPKI: *App*^*NL-G-F/NL-G-F*^. **(B)** Atlas coregistration to group template. **(C)** Functional connectome construction. Time series from each voxel of the hippocampus was extracted using the atlas look-up table. The Pearson correlation of the time series between voxels was used to create the functional connectivity matrix for each subject.

Two-step registration was performed to coregister subject EPI data to the same template space (Figure 1B). A wild-type mouse from the middle age group was arbitrarily chosen as the group template. Briefly, each subject's mean EPI image was registered to their respective *T*_2_-weighted image. Then, the *T*_2_-weighted image was registered to the group template *T*_2_, and the transformation matrices for each step were applied to each subject's EPI image. Detailed coregistration steps are given in the Supplementary material.

#### 2.3.2. Atlas label registration

The high-resolution *T*_2_-weighted mouse template image and atlas labels used were made by Johnson et al. (2010) and Ullmann et al. (2013). When using the labels defined by Ullmann et al., the CA2 was combined with the CA3 due to its small area. The high-resolution *T*_2_-weighted mouse template image was registered to the group template using FSL's flirt tool with 12 degrees of freedom. The transformation matrix for this step was then applied to the atlas labels using nearest neighbor interpolation.

#### 2.3.3. Connectome construction

The atlas label NIfTI file was used to look up voxel indices for the hippocampus and its subregions. For voxel-wise connectomes, the time series from each hippocampus voxel for each subject was arranged in an *N* × *T* × *S* array containing the BOLD time series for all *N* voxels at *T* time points across *S* subjects. The Pearson correlation was computed between all pairwise voxels to create the *N* × *N* voxel-wise correlation matrices. For hippocampal subregion correlation matrices, the time series within each hemisphere subregion was averaged. The Pearson correlation of the averaged time series between all pairwise subregions was calculated to create the subregion correlation matrices.

### 2.4. Statistical analysis

#### 2.4.1. Interhemispheric voxel-wise analysis

Statistical analyzes were performed using R version 3.6.3 (2020-02-29) (R Core Team, 2018). For voxel-wise interhemispheric analyses, the voxels for left–right correlations were first extracted from each subject's functional connectome (i.e., the off-diagonal block of the functional connectome). The mean correlation value of these voxels was calculated as the observation for each subject. A two-way Type II ANOVA was fit to model the correlation by age group and genotype according to the equation


(1) $$r_{i}=b_{0}+b_{1}{\text{age}}_{i}+b_{2}{\text{genotype}}_{i}+b_{3}{\left(\text{age}\times \text{genotype}\right)}_{i}+\varepsilon_{i},$$

for subjects *i* = {1, …, *S*}, where age (early, middle, late) and genotype (wild-type, APPKI) were treated as categorical variables. The afex (Singmann et al., 2021) and emmeans (Lenth, 2021) packages in R were used to model this as aov_car(r ~ age * genotype + Error(subject)). *Post hoc* comparisons for age group were performed using Fisher's LSD.

#### 2.4.2. Interhemispheric subregion analysis

Linear mixed models (LMMs) were fit using the lme4 (Bates et al., 2015) and lmerTest (Kuznetsova et al., 2017) packages. For interhemispheric correlation by subregion analyses, the Pearson correlation between the average time series for each subregion pair was modeled by


(2) $$\begin{array}{l} r_{ij}=(b_{0}+u_{0j})+\left(b_{1j}+u_{1j}\right){\text{age}}_{ij}+b_{2}{\text{genotype}}_{ij}\\ \;+b_{3}{\left(\text{age}\times \text{genotype}\right)}_{ij}+\varepsilon_{ij}, \end{array}$$

for subjects *i* = {1, . . . , *S*} and regions *j* = {1, . . . , *N*}, where *u*_0*j*_ specifies a random intercept for the *j*th region and *u*_1*j*_ specifies a random slope for age for the *j*th region. In lme4, this was fit using lmer(r ~ age * genotype + (1 + age | region_pair)).

#### 2.4.3. Intrahemispheric analysis

For intrahemispheric correlation analyzes, the linear mixed model in Equation 2 was extended to include hemisphere as a fixed effect to give the following model


(3) $$\begin{array}{l} r_{ijk}=\left(b_{0}+u_{0jk}\right)+\left(b_{1}+u_{1jk}\right){\text{age}}_{ijk}+b_{2}{\text{genotype}}_{ijk}\\ \;+b_{3}{\text{hemisphere}}_{ijk}+b_{4}{\left(\text{age}\times \text{genotype}\right)}_{ijk}\;\\ \;+b_{5}{\left(\text{genotype}\times \text{hemisphere}\right)}_{ijk}\\ \;+b_{6}{\left(\text{age}\times \text{hemisphere}\right)}_{ijk}\\ \;+b_{7}{\left(\text{age}\times \text{genotype}\times \text{hemisphere}\right)}_{ijk}+\varepsilon_{ijk}, \end{array}$$

for subjects *i* = {1, . . . , *S*}, regions *j* = {1, . . . , *N*}, and hemispheres *k* = {1, 2}, allowing a random intercept for each subregion pair nested within hemisphere and random slope for age within each subregion pair and hemisphere. The fixed effects tested were age, genotype, and hemisphere. In lme4, this was modeled as lmer(r ~ age * genotype * hemisphere + (1 + age | hemisphere/subregion_pair)).

For all LMMs, age (in months) was treated as a continuous variable and centered to the mean of the early age group (4.57 months). Effect sizes for effects with two levels were calculated as described by Westfall et al. (2014) and Brysbaert and Stevens (2018), where the effect size *d*_*i*_ of the *i*th fixed effect was calculated as


(4) $$\begin{array}{l} d_{i}=\frac{\mu_{i,1}-\mu_{i,2}}{\sqrt{\underset{j}{\sum }\sigma_{j}^{2}}}=\frac{\beta_{i}}{\sqrt{\underset{j}{\sum }\sigma_{j}^{2}}}, \end{array}$$

where *μ*_*i*,1_, *μ*_*i*,2_ are the means of the two levels of the *i*th fixed effect, *β*_*i*_ is the estimate of the fixed effect from the model, and $\sigma_{j}^{2}$ is the variance of the *j*th random effect for each random effect variable in the model. The centered age values were transformed to their original values for clarity in graphics.

### 2.5. Software

Neuroimaging processing and analysis was performed using a combination of SPM12 (SPM, 2017) and CONN (Whitfield-Gabrieli and Nieto-Castanon, 2012) with MATLAB version R2017b (MathWorks, 2017), and FSL version 6.0 (Jenkinson et al., 2012) using Nipype (Gorgolewski et al., 2011) in Python version 3.8 from the Anaconda distribution (Anaconda, 2018) with associated scientific computing libraries (Jones et al., 2001; McKinney, 2010; Pedregosa et al., 2011; Brett et al., 2020; Harris et al., 2020; Waskom, 2021). Visualization was done using ggplot2 (Wickham, 2016), matplotlib (Hunter, 2007), and seaborn (Waskom, 2021). Inkscape version 0.92 (Ink, 2017) and GNU Image Manipulation Program version 2.8.16 (Kimball et al., 2016) were used for arrangement of figures.

## 3. Results

### 3.1. Increased interhemispheric connectivity in the hippocampus with age

**Figure 2:**
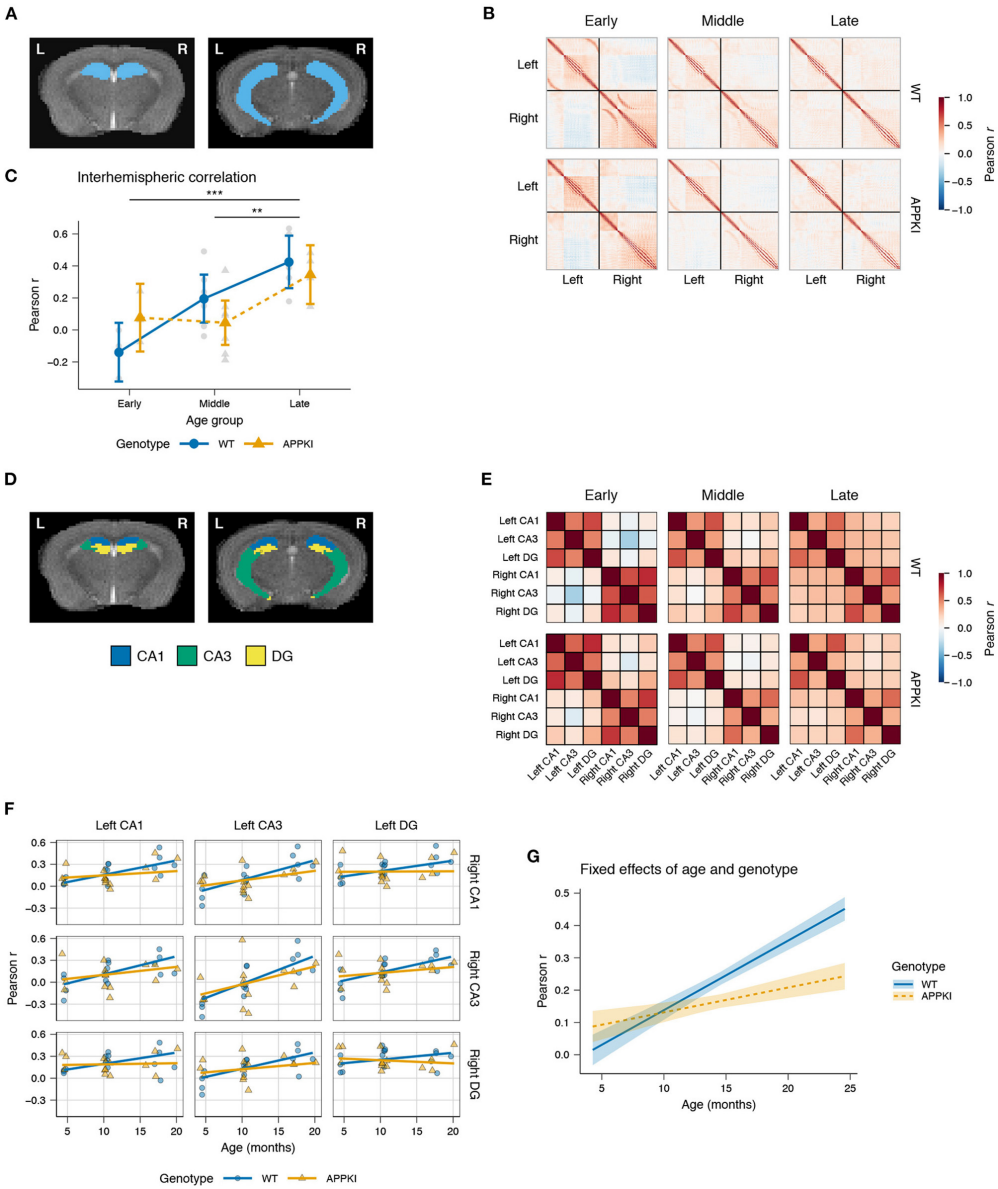
Hippocampal interhemispheric correlation by age and genotype. **(A)** Representative *T*_2_-weighted images of coronal slices containing the hippocampus. **(B)** Group average Pearson correlation matrices for each voxel in the hippocampus. Black lines demarcate the left and right hemispheres (upper left and lower right, respectively). **(C)** Lineplot of the interhemispheric Pearson correlation by age group and genotype. Gray points represent the mean interhemispheric FC for each subject. Colored points represent group mean. Error bars represent 95% CI. **(D)** Representative atlas images of the hippocampal CA1, CA3, and DG. **(E)** Pearson correlation matrices of the average time series for each subregion, averaged across group. **(F)** Linear mixed model fit of interhemispheric correlation by age and genotype, with a random intercept for each subregion and a random slope for age within subregion. Points represent mean interhemispheric FC for each subject. Lines represent model fit. **(G)** Fixed effects of age and genotype. Lines represent model fit. Shaded areas represent standard error. Blue: wild-type; orange: *App*^*NL-G-F/NL-G-F*^. ***p < 0.001; **p < 0.01.

In order to study the effects of aging and familial Alzheimer's disease (FAD) mutations on hippocampal connectivity, we performed *in vivo* imaging of wild-type (WT) and *App*^*NL-G-F/NL-G-F*^ knock-in (APPKI) female mice using a 9.4 T MRI. We acquired anatomical and rs-fMRI images of mice in early (4 months), middle (10 months) and late (>15 months) age groups and constructed the functional connectome of the hippocampus for each mouse (Figure 1; representative image of the hippocampus is highlighted in Figure 2A). First, we asked whether there were overall changes in the connectivity between hemispheres with age. We extracted the interhemispheric voxels from each subject's connectome to test if there were changes in the mean interhemispheric correlation across age groups (Figure 2B). Two-way ANOVA results showed a significant main effect of age [*F*_(2,23)_ = 12.6878, *p* < 0.0001] (Figure 2C). *Post hoc* contrasts revealed statistically significant differences between early–late [Fisher's LSD, *t*_(23)_ = −4.621, *p* = 0.0001] and middle–late age groups [Fisher's LSD, *t*_(23)_ = −3.430, *p* = 0.0023]. Interestingly, we observed that the early APPKI group had a higher correlation comparable to the middle APPKI group, whereas the wild-type group had a lower correlation in the early group that exceeded the middle APPKI group. Thus, across the whole hippocampus, the correlation between hemispheres increased with age.

### 3.2. Higher interhemispheric connectivity between hippocampal subregions, followed by later decrease, in *App^NL-G-F/NL-G-F^* compared to wild-type mice

Next, we examined the individual subregions of the hippocampus to test whether there were subregion-specific changes in interhemispheric connectivity. We used the atlas by Ullmann et al. (2013) to obtain the hippocampal subregion divisions for the *cornu Ammonis* (CA) fields 1 and 3 (CA1, CA3, respectively) and the dentate gyrus (DG; Figure 2D). We calculated the average time series within each subregion and computed the pairwise Pearson correlation between time series for each subject (Figure 2E). A linear mixed model (LMM) was used to fit the correlation by age and genotype within each pair of subregions, allowing the slope and intercept to be variable to account for intrinsic differences in connectivity within each pair of subregions.

Among the left–right pairs of subregions, there was relatively little change in interhemispheric connectivity across age with the DG, and moderate increase in the CA1, while the left–right CA3 was found to have the largest increase in correlation with age (Figure 2F). Overall, we observed a statistically significant interaction between age and genotype [*t*_(252)_ = −3.837, *p* < 0.001; Figure 2G]. The fixed effects of age and genotype, controlling for the intrinsic differences across subregion pairs, showed an increase in interhemispheric connectivity for APPKI mice in early age, with a notably reduced interhemispheric connectivity after middle age relative to the wild-type. Finally, we observed that the effect size for genotype was *d* = 0.363, suggesting that this was a moderately-sized effect. Together, these data suggest that there is higher hippocampal connectivity between hemispheres in early APPKI mice compared to wild-type, but after middle age the APPKI show reduced interhemispheric connectivity compared to wild-type.

### 3.3. Loss of laterality in intrahemispheric connectivity in *App^NL-G-F/NL-G-F^* mice

After observing a consistently increased correlation between the left–right hippocampus across age, we next asked whether there was a converse decrease in intrahemispheric connectivity across age, and whether this decrease was consistent for both hemispheres. To do this, we similarly fit a LMM using age, genotype, and hemisphere as fixed effects, while allowing the slope for age to be variable within each subregion pair nested within each hemisphere and allowing the intercepts to be variable for each subregion pair.

**Figure 3:**
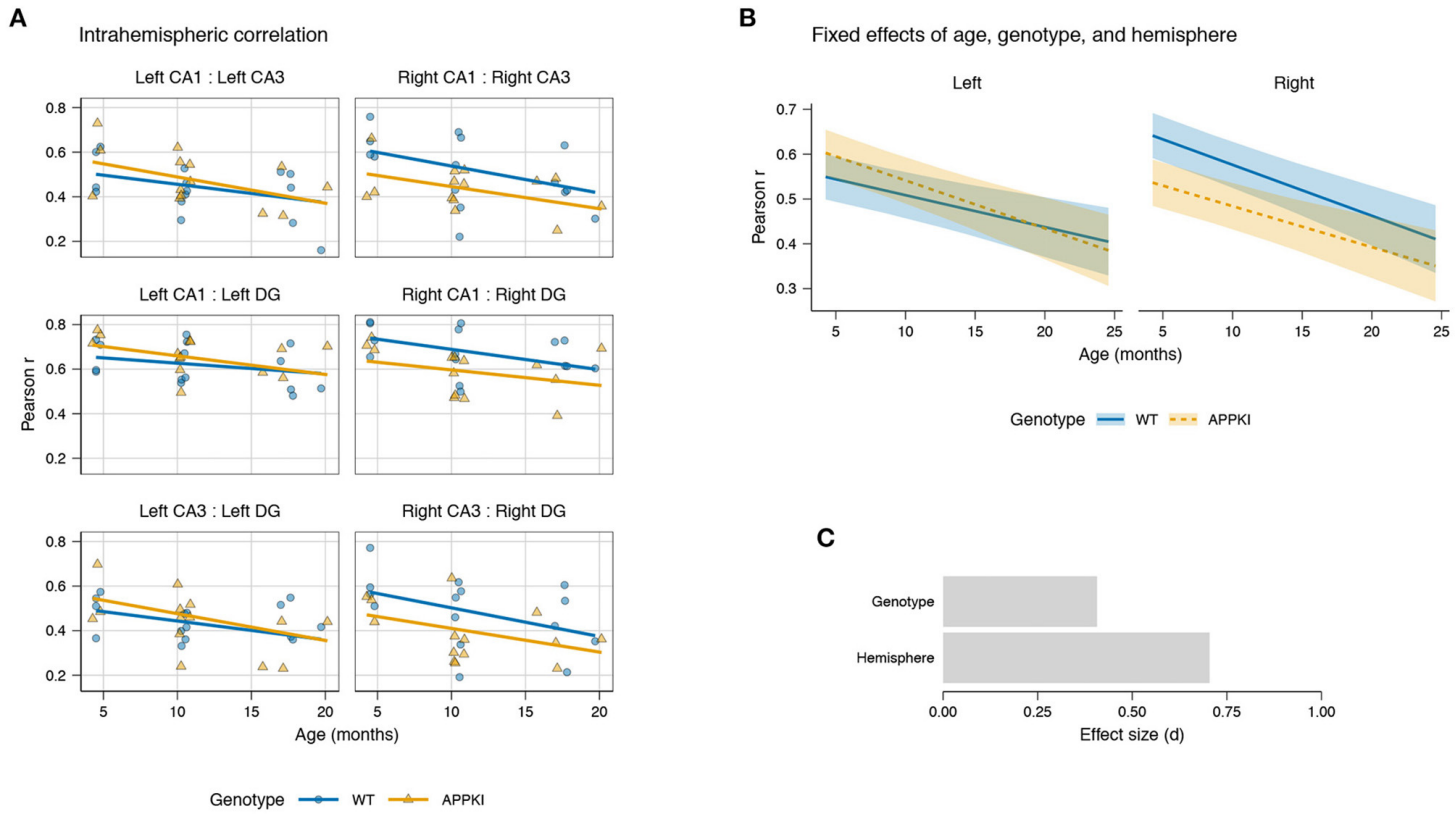
Hippocampal intrahemispheric correlation by age and genotype. **(A)** Linear mixed model fit for intrahemispheric correlation between subregions for CA1, CA3, and DG in each hemisphere. Points represent mean intrahemispheric FC for each subject. Lines represent model fit. **(B)** Fixed effects of age, genotype, and hemisphere on correlation. Lines represent model fit. Shaded areas represent standard error. Blue: wild-type; orange: *App*^*NL-G-F/NL-G-F*^. **(C)** Effect sizes of genotype and hemisphere on correlation.

Overall, we observed decreasing intrahemispheric correlation with age across both genotypes for each pair of subregions (Figure 3A). LMM results showed a statistically significant fixed effect of age [*t*_(7.88)_ = −2.283, *p* = 0.0230] and a statistically significant interaction between genotype and hemisphere [*t*_(168)_ = −2.943, *p* = 0.00371; Figure 3B]. Among the pairs of subregions, we observed the greatest decrease in correlation with age in the CA3–DG for both hemispheres, followed by CA1–CA3, and CA1–DG. The effect size for genotype was 0.407 and for hemisphere was 0.704 (Figure 3C).

While both hemispheres showed decreasing intrahemispheric connectivity across age, the effect was different between the left and right hemispheres, where the wild-type had a higher correlation in the right hemisphere compared to the left (Figure 3B). Furthermore, we observed a genotype difference primarily in the right hemisphere, where the APPKI mice showed reduced correlation compared to wild-type, especially in early age. Together, this suggests that there is a lateral asymmetry in intrahemispheric connectivity in the wild-type mice that is not present in the APPKI, with the right hemisphere having an increased correlation relative to the left hemisphere.

## 4. Discussion

In this study, we observed age-related alterations in FC of the hippocampus in the *App*^*NL-G-F/NL-G-F*^ mouse model of AD. Evidence from previous studies suggests that functional and structural interhemispheric connectivity is disrupted in AD compared to healthy aging controls (Lakmache et al., 1998; Wang et al., 2015; Li et al., 2018), as well as reduced network connectivity (Allen et al., 2007), particularly in the default mode network (DMN) (Raichle et al., 2001; Greicius et al., 2004), and atrophy of major white matter fibers, such as the corpus callosum (Li et al., 2018). A previous study by Wang et al. (2015) found that interhemispheric FC is reduced in AD patients, and was correlated with reduced integrity of the corpus callosum measured via DTI, as well as correlated with reduced cognitive scores. Further, it has been observed that disconnection of the corpus callosum, e.g., for the treatment of epilepsy, leads to significant loss of interhemispheric FC (Johnston et al., 2008; Wang et al., 2015). Since myelin and oligodendrocytes are also known to be affected in AD (Bartzokis, 2011), this suggests that structural integrity of white matter connections may also be important for preservation of functional connectivity. Many AD patients also experience disruptions in their circadian rhythm (Webster et al., 2014), and interhemispheric connectivity can also be modulated by sleep deprivation and changes in circadian rhythm (Zhu et al., 2016). While the precise mechanisms are still not well understood, taken together, this suggests that there is a complex interaction between brain structure, function, and environmental factors in AD pathology.

This reduction in resting-state interhemispheric and DMN connectivity in AD may be coupled with a complementary increase in task-induced activity. With altered resting-state connectivity, it may be more difficult for the brain to inhibit activity not relevant for a given task (Celone et al., 2006; Sheline and Raichle, 2013), referred to as task-induced deactivation (TID). Indeed, previous studies have shown that more cognitively demanding tasks are also associated with higher TID (Daselaar et al., 2004). Early MCI patients showed increased hippocampal activity during an episodic memory task and less TID relative to controls (Celone et al., 2006), in this context referred to as hippocampal “hyperexcitation.” The study by Bakker et al. (2012) observed hyperexcitation in the CA3/DG in mild cognitive impairment (MCI) patients relative to controls during memory-related tasks. Treatment with anti-epileptic medication, which effectively reduced the hyperactivity, resulted in increased memory performance in these patients (Bakker et al., 2012, 2015). Furthermore, hippocampal hyperexcitation has been shown to precede the onset of the cognitive symptoms. Analysis of the hyperexcitation index (HI) in cognitively healthy middle-aged APOE4 carriers and non-carriers showed that female APOE4 carriers have an increased HI relative to non-carriers (Fortel et al., 2020).

At the neurobiological level, this may be due to induced neuronal expression of APOE, leading to APOE4-mediated toxicity of GABAergic interneurons in the hippocampus, which could lead to wider network hypersynchrony (Najm et al., 2019). While the majority of GABAergic neurons synapse locally, previous studies have identified long-range GABAergic projections, in the hippocampal commissure from the hilus and from the CA3 and CA1 (Ribak et al., 1986). Notably, in the hAPP-J20 mouse model, which expresses human APP with FAD mutations (Mucke et al., 2000), imbalance between GABAergic and glutamatergic transmission has been reported to compromise hippocampal neurogenesis, which regulates the activity level of the DG and hippocampus (Sun et al., 2009). In addition, evidence from mouse stereological studies suggests that the DG, CA1, and CA3 each have specific GABAergic to glutamatergic neuron ratios, with ≈1.5 % in the DG, ≈11 % in the CA1, and ≈10 % in the CA3 in the dorsal hippocampus. In the ventral hippocampus, the GABAergic ratio is larger, with ≈4 % in the DG, ≈22 % for the CA1, and ≈21 % for the CA3. Thus, the CA1 and CA3 have comparable GABAergic ratios, while the DG has the lowest (Jinno et al., 1998; Jinno and Kosaka, 2009). Furthermore, previous studies suggest that there may be age-related changes in both glutamatergic and GABAergic signaling with age and AD (Stephens et al., 2011; Albuquerque et al., 2015; Hollnagel et al., 2019; Kumar and Foster, 2019). Here, we observed larger changes in interhemispheric FC with age primarily in the CA3 and CA1 subregions. Given that the CA1 and CA3 have a relatively larger GABAergic ratio relative to the DG, this may suggest that there are alterations in the excitation-inhibition balance in these regions. Consistent with this, a recent study by Arroyo-García et al. (2021) reported disruption in γ oscillations in fast-spiking interneurons in the CA3 of *App*^*NL-G-F/NL-G-F*^ mice by 2 months of age, prior to the onset of amyloid plaque formation. They also observed that γ oscillations in CA3 pyramidal cells become disrupted by 6 months of age, the age at which Saito et al. (2014) had previously observed cognitive impairments to begin. This suggests that these early changes in excitation-inhibition balance may be due to soluble A*β*. Additionally, the septum, an important regulator of hippocampal θ oscillations, as well as the entorhinal cortex, contain long-range GABAergic connections with the hippocampus. These long-range projections also primarily synapse to other GABAergic neurons, suggesting that they may function to regulate synchronous activity in the hippocampus (Caputi et al., 2013). Thus, changes in the functional connectivity in the hippocampus in AD may be due in part to disinhibition of GABAergic regulatory circuits that lead to hyperexcitation-induced toxicity (Najm et al., 2019).

The inter/intrahemispheric FC changes observed may be influenced by the neurobiological asymmetries known to exist in the hippocampus (Jordan, 2020). For example, there is left–right asymmetric expression of synaptic receptors. Left ipsi- and contralateral CA3–CA1 projections primarily express the GluN2B receptor, whereas the right ipsi- and contralateral CA3–CA1 projections express the GluR1 receptor (Kawakami et al., 2003; Shinohara et al., 2008; Jordan, 2020). Consequently, these projections have distinct synaptic plasticity properties. These asymmetries also extend to the behavioral level. Based on data from previous studies, the discrete–continuous model of spatial processing suggests that the left CA3 may encode salient features, whereas the right CA3 processes more continuous navigation (Jordan, 2020). These studies highlight that there are molecular and functional asymmetries in the hippocampus and throughout the brain, which may be important for cognitive performance. For example, a study by Shimbo et al. (2018) showed that knockout of the *β*2-microglobulin, which helps to establish hippocampal circuit left-right asymmetry (Kawahara et al., 2013; Shimbo et al., 2018), resulted in slower learning of non-spatial cognitive tasks compared to control mice, suggesting that intact asymmetry is important for healthy cognitive function. In humans, Lakmache et al. (1998) observed that AD patients engaging in tasks requiring interhemispheric connectivity performed worse compared to healthy control patients, while tasks requiring only one hemisphere were not nearly as impaired. Together, the combined effects of aging and AD pathology may be responsible for reducing these functional asymmetries, leading to reduced memory performance and cognitive deficits seen in AD patients.

Resting-state fMRI has become increasingly used for studying rodent models (Mandino et al., 2020). Previous studies in *App*^*NL-G-F/NL-G-F*^ and *App*^NL-F/NL-F^ mouse models have observed increased bilateral hippocampal activity (Shah et al., 2018) and prefrontal synchrony (Latif-Hernandez et al., 2019) around 3–4 months of age, with subsequent decline after 7 months of age, which is consistent with our observations, suggesting that between 4 and 6 months of age there is a shift from increased to decreased interhemispheric connectivity in the hippocampus. Around this age point, 5×FAD mice have also been shown to have alterations in their functional connectome network (Kesler et al., 2018). In older age groups, reduced hippocampal FC has been observed by 10 months of age in longitudinal studies of TgF344 (APPSwe; PSEN1ΔE9) rats (Anckaerts et al., 2019), and reduced interhemispheric FC was reported in 18-month-old APP/PS1 mice (Shah et al., 2013). Reduced FC has also been observed in aged APOE mouse models (Zerbi et al., 2014), suggesting that disruptions in FC observed in mouse models with APP and presinilin FAD mutations may also generalize to other AD genetic risk factors. Together, these studies and ours suggest that in early age there is higher functional activity in AD rodent models that decreases around middle age and continues with increasing age, and appears in both sexes and in mouse models with multiple AD-related mutations.

There are some limitations to this study. While only female mice were used in this study, this was translationally relevant in that females are significantly more likely to develop AD (Li and Singh, 2014; Alzheimer's Association, 2022), as well as controls for sex-specific differences in the current study. However, future studies with male mice will be needed to determine if the functional dynamics we observed in female mice may be different in male mice. Finally, there are also potential confounds of anesthesia that should be considered, since this is a crucial distinction between human and animal rs-fMRI. Previous studies have described the effects of various anesthetics on functional networks in the context of animal imaging (Bukhari et al., 2017; Mandino et al., 2020). While consistency of isoflurane administration is taken, it is possible there are variable effects of isoflurane on neurovascular coupling with aging or between different individuals. With this caveat in mind, however, while higher concentrations of isoflurane may impact interhemispheric connectivity (Mandino et al., 2020), previous studies have shown that interhemispheric FC is preserved with low doses of isoflurane comparable to that observed in awake mice (Jonckers et al., 2014; Zerbi et al., 2014).

## 5. Conclusion

In summary, we used *in vivo* resting-state fMRI to measure the FC of wild-type and *App*^*NL-G-F/NL-G-F*^ mice in different age groups. We observed higher interhemispheric FC, followed by later decrease in interhemispheric FC in the hippocampus of *App*^*NL-G-F/NL-G-F*^ mice compared to wild-type mice. Furthermore, we observed no laterality in intrahemispheric FC in the *App*^*NL-G-F/NL-G-F*^ mice. Together, these results suggest that FC in the context of AD is affected by both aging and hemispheric asymmetry, and that early alterations in hippocampal activity may be an important biomarker and for understanding the progression of the disease.

## Data availability statement

The raw data supporting the conclusions of this article will be made available by the authors, without undue reservation.

## Ethics statement

The animal study was reviewed and approved by Animal Care Committee (ACC) at the University of Illinois at Chicago.

## Author contributions

ZM, OL, and AL designed research. ZM, JG, and WL performed research. TSaid and TSait contributed unpublished reagents/analytic tools. ZM, LZ, IF, and AL analyzed data. ZM wrote the paper. ZM, JG, LZ, WL, IF, AB, SM, OA, OL, and AL edited the paper. All authors contributed to the article and approved the submitted version.
