# Supplementary material for: Hippocampal functional connectivity across age in an App knock-in mouse model of Alzheimer's disease
Source: Front Aging Neurosci. 2023 Jan 12;14:1085989. doi: 10.3389/fnagi.2022.1085989 (PMC9878347; doi:10.3389/fnagi.2022.1085989)
Supplement: Supplementary file 1 [file Data_Sheet_1.pdf]

Supplementary Material

1 COREGISTRATION DETAILS

1.1 Intra-subject registration.

Two-step registration was performed to coregister subject EPI data to the same template space. A wild-type mouse from the middle age group was arbitrarily chosen as the group template. To improve coregistration accuracy, each subject's mean EPI image was downsampled from $256 \times 256 \times 2$ voxels to $128 \times 128 \times 2$ voxels to match the resolution of their corresponding T_2-weighted image. The downsampled slices were then copied to an empty array of the same dimension as their corresponding T_2-weighted image, and the z-index for each EPI slice was matched to its corresponding T_2-weighted image z-index. A mask of the brain was created manually using FSL's `fsleyes` and then applied to each downsampled mean EPI image using `fslmaths`. A coplanar T_2-weighted image was created using the two T_2-weighted coronal slices that were coplanar to the EPI slices, with all other slices set to zero. A 2D rigid coregistration was then performed using FSL's `flirt` tool to align each subject's EPI to their T_2-weighted image and the transformation matrix T_1 was saved.

1.2 EPI to group template registration.

The T_2-weighted image for each subject was registered to the group template T_2-weighted image using FSL's `flirt` tool with 9 degrees of freedom. The resulting transformation matrix T_2 was saved. The transformation matrices T_1 and T_2 (corresponding to subject EPI $\rightarrow$ subject T_2-weighted coplanar, and subject T_2-weighted image $\rightarrow$ group template, respectively) were then concatenated and applied to each subject's 4D EPI data to register to the group template.

1.3 Coregistration quality assurance.

The EPI data quality and coregistration accuracy were checked for each subject. Subjects with poor coregistration results or excessive artifacts in the EPI image were removed. To quantitatively measure goodness-of-fit of each subject to the group template, the mutual information (MI) between the coregistered T_2-weighted coplanar image and the group template T_2-weighted image was calculated from the joint histogram between the images. The mean $\pm$ SD was $\mathrm{MI} = 0.83 \pm 0.09$ (see Table S1 for details).

Table S1. Mutual information of coregistered subject T_2-weighted images with group template T_2-weighted image.

Subject	Genotype	Age group	Age (months)	MI with template
sub-02	APPKI	early	4.80	0.89
sub-03	WT	early	4.50	0.81
sub-04	WT	early	4.80	0.82
sub-06	APPKI	middle	10.84	0.84
sub-07	APPKI	middle	10.87	0.68
sub-09	WT	middle	10.25	0.82
sub-10	WT	middle	10.28	0.85
sub-12	WT	early	4.50	0.82
sub-14	WT	early	4.50	0.89
sub-16	APPKI	early	4.27	0.87
sub-18	APPKI	middle	10.02	0.76
sub-20	WT	middle	10.48	0.86
sub-23	APPKI	early	4.60	0.83
sub-24	APPKI	middle	10.15	0.88
sub-25	APPKI	middle	10.19	0.75
sub-26	APPKI	middle	10.22	0.80
sub-27	APPKI	middle	10.25	0.85
sub-28	WT	middle	10.55	0.81
sub-31	WT	middle	10.64	1.17
sub-36	APPKI	late	15.77	0.79
sub-38	WT	late	16.99	0.83
sub-40	APPKI	late	17.05	0.87
sub-41	APPKI	late	17.15	0.84
sub-43	APPKI	late	20.14	0.87
sub-44	WT	late	19.71	0.89
sub-46	WT	late	17.64	0.81
sub-47	WT	late	17.68	0.73
sub-49	WT	late	17.81	0.64

2 LINEAR MIXED MODEL TABLES

The following tables contain results for linear mixed models used in this study.

Table S2. Descriptive statistics of mouse subjects scanned using MRI. The number of mice (n) and mean and standard deviation (SD) age in months is displayed.

Genotype	Age group	Sex	n	Age (months)	
				Mean	SD
Wild-type	Early	♀	4	4.58	0.15
	Middle	♀	6	10.47	0.17
	Late	♀	5	17.97	1.03
App$^{NL\text{-}G\text{-}F/NL\text{-}G\text{-}F}$	Early	♀	3	4.56	0.27
	Middle	♀	7	10.36	0.35
	Late	♀	4	17.53	1.85

Table S3. Descriptive statistics for interhemispheric correlation by age and genotype.

Genotype	Age group	n	Mean	SD
WT	Early	4	−0.139	0.125
	Middle	6	0.196	0.195
	Late	5	0.425	0.19
App$^{NL\text{-}G\text{-}F/NL\text{-}G\text{-}F}$	Early	3	0.077	0.159
	Middle	7	0.045	0.193
	Late	4	0.345	0.148

Table S4. Two-way Type II ANOVA results for hippocampal interhemispheric correlation by age and genotype. Model: $r_i = b_0 + b_1\text{age}_i + b_2\text{genotype}_i + b_3\,(\text{age} \times \text{genotype})_i + \varepsilon_i$. In `afex`: `aov_car(r ~ age_group * genotype + Error(subject))`. Significance codes: ***$p < 0.001$, **$p < 0.01$, *$p < 0.05$.

Term	df_1	df_2	MSE	F	η^2_g	p	sig.
Age	2	23	0.031	12.688	0.525	0.0002	***
Genotype	1	23	0.031	0.384	0.016	0.5410	
Age × Genotype	2	23	0.031	2.467	0.177	0.1070	

Table S5. *Post hoc* contrasts for main effect of age for interhemispheric ANOVA in Table S4.

Contrast	Estimate	SE	df	t ratio	p	Sig.
Early–Middle	−0.151	0.0838	23	−1.808	0.0838	·
Early–Late	−0.416	0.0901	23	−4.621	0.0001	***
Middle–Late	−0.265	0.0772	23	−3.430	0.0023	**

Table S6. Linear mixed model fixed effects for hippocampal interhemispheric correlation for each subregion. Model: $r_{ij} = (b_0 + u_{0j}) + (b_1 + u_{1j})\,\text{age}_{ij} + b_2\text{genotype}_{ij} + b_3\,(\text{age} \times \text{genotype})_{ij} + \varepsilon_{ij}$, for $i, \ldots, S$ subjects and $j, \ldots, N$ regions. In `lme4`: `lmer(r ~ age_months * genotype + (1 + age_months | region))`. AIC = −235.3, BIC = −206.8, LL = 125.7, $\text{df}_{\text{resid}} = 253$. Significance codes: ***$p < 0.001$, **$p < 0.01$, *$p < 0.05$.

Fixed effects	Estimate	Std. Error	df	t	p	sig.
Intercept	0.021	0.046	10.856	0.463	0.6530	
Age	0.022	0.004	14.278	5.897	< 0.0001	***
Genotype	0.068	0.030	252.000	2.274	0.0240	*
Age × Genotype	−0.014	0.004	252.000	−3.837	0.0002	***

Table S7. Linear mixed model random effects for hippocampal interhemispheric correlation for each subregion from Table S6.

Groups	Name	Variance	SD
Region	Intercept	1.562×10^{-2}	0.124988
	Age	6.949×10^{-5}	0.008336
Residual		2.064×10^{-2}	0.143664

Table S8. Linear mixed model random effects for hippocampal interhemispheric correlation for each subregion from Table S7.

Region		Intercept	Slope (Age)
Left	Right		
CA1	CA1	0.027	−0.002
	CA3	−0.049	0.003
	DG	0.092	−0.006
CA3	CA1	−0.082	0.005
	CA3	−0.253	0.017
	DG	−0.014	0.001
DG	CA1	0.107	−0.007
	CA3	−0.008	0.001
	DG	0.180	−0.012

Table S9. Intrahemispheric LMM results. AIC = −245.2, BIC = −197.8, LL = 137.6, Deviance = −275.2, $\text{df}_{\text{resid}} = 159$. Model was fit in `lme4` as `lmer(r ~ age * genotype * hemisphere + (1 + age | hemisphere/subregion))`. Significance codes: ***$p < 0.001$, **$p < 0.01$, *$p < 0.05$.

Fixed effects	Estimate	Std. Error	df	t	p	Sig.
Intercept	0.547221	0.050308	7.875136	10.877	< 0.00001	***
Age	−0.007120	0.003119	43.194936	−2.283	0.02742	*
Genotype	0.052438	0.037663	168.000105	1.392	0.16567	
Hemisphere	0.090768	0.071147	7.875136	1.276	0.23837	
Age × Genotype	−0.003594	0.004527	168.000105	−0.794	0.42827	
Age × Hemisphere	−0.004247	0.004411	43.194936	−0.963	0.34097	
Genotype × Hemisphere	−0.156741	0.053263	168.000105	−2.943	0.00371	**
Age × Genotype × Hemisphere	0.005817	0.006402	168.000105	0.909	0.36480	

Table S10. Intrahemispheric LMM random effects from Table S9.

Groups	Name	Variance	SD
Hemisphere/region	Intercept	5.641×10^{-3}	7.511×10^{-2}
	Age	2.835×10^{-6}	1.684×10^{-3}
Hemisphere	Intercept	3.653×10^{-10}	1.911×10^{-5}
	Age	2.142×10^{-14}	1.463×10^{-7}
Residual		1.084×10^{-2}	1.041×10^{-1}

Table S11. Intrahemispheric LMM random effects for each subregion from Table S9.

Hemisphere	Region	Intercept	Slope (Age)
Left	CA1–CA3	−0.04698644	−0.0010533816
	CA1–DG	0.10524476	0.0023594658
	CA3–DG	−0.05825832	−0.0013060842
Right	CA1–CA3	−0.03419498	−0.0007666118
	CA1–DG	0.10021060	0.0022466056
	CA3–DG	−0.06601562	−0.0014799938
